# Examining the Cross-Cultural Sensitivity of the Revised Two-Factor Study Process Questionnaire (R-SPQ-2F) and Validation of a Dutch Version

**DOI:** 10.1371/journal.pone.0054099

**Published:** 2013-01-16

**Authors:** Ann Stes, Sven De Maeyer, Peter Van Petegem

**Affiliations:** Institute for Education and Information Sciences, University of Antwerp, Antwerp, Belgium; Universidad Europea de Madrid, Spain

## Abstract

The Revised Two-Factor Study Process Questionnaire (R-SPQ-2F) is used to examine students’ study approaches in higher education. The questionnaire assumes to measure two factors: a deep and a surface study approach. Analyses into the validity and reliability of the original English R-SPQ-2F yielded positive results. In this study, we examined the degree to which these positive results can also be found for the Dutch version that we developed. By comparing our results with the results of earlier studies in different cultures, we conclude cross-cultural sensitivity is an important point to be borne in mind when using the R-SPQ-2F. Our research supports the validity and reliability of our Dutch version of the R-SPQ-2F.

## Introduction

The Revised Two-Factor Study Process Questionnaire (R-SPQ-2F) [1] has frequently been used to examine the study approaches of students in higher education in different countries and subject areas. In the original version of the Study Process Questionnaire (SPQ) [2] distinction was made between three approaches to learning (surface, deep and achieving), each with a motive and a strategy subscale. However, studies into the dimensionality of the SPQ [3], [4] indicated a two factor solution with surface and deep approaches had the best fit. In line with the findings of these studies as well as because of the changed nature of higher education (e.g. new teaching and assessment methods, increased heterogeneity of student population) Biggs, Kember and Leung [1] revised the SPQ. The revised version (R-SPQ-2F) is a short questionnaire (20 items, scored on a 5 point Likert scale), and is easily to use. It categorises student’s learning approach as surface when the student experiences learning as an external duty necessary to pass a course and when he/she tries to meet the requirements of courses with a minimum effort; student’s learning approach is categorised as deep when the student has an intrinsic interest in learning and expects that he/she will enjoy learning. Both scales (Surface Approach, Deep Approach) contain two subscales: the subscale ‘Strategy’ is about the way a student goes about his/her study; the subscale ‘Motive’ is about the reasons for adopting a strategy.

In their study of 2001, Biggs, Kember, and Leung analysed the psychometric properties of the revised version of the questionnaire based on data from 495 undergraduate students from various disciplines and across each year of study at a university in Hong Kong. A confirmatory factor analysis supported the unidimensionality of the four subscales, with Cronbach’s alpha values of 0.62, 0.63, 0.72 and 0.57 for the Deep Motive factor, Deep Strategy factor, Surface Motive factor and Surface Strategy factor, respectively. With respect to the two main scales Cronbach’s alpha values of 0.73 and 0.64 were reported for the Deep Approach factor and Surface Approach factor, respectively.

Two hypothesized models were tested. A first model looked at the structure of the questionnaire from the items level and consisted of the four subscales formulated as latent constructs with their corresponding five items as indicators. A confirmatory factor analysis supported that the items are good indicators of the four constructs. High correlations were found between the deep motive and deep strategy subscales on the one hand, and between the surface motive and surface strategy subscales on the other hand. A second model further tested the dimensionality of the instrument by treating the four subscales as indicators of the two main scales formulated as latent constructs. A confirmatory factor analysis supported the supposed dimensionality. As expected the Deep Approach construct and Surface Approach construct were negatively related. Biggs, Kember, and Leung concluded that these results provide the psychometric quality of the R-SPQ-2F [1]. The questionnaire can be used in a two-factor form, distinguishing deep and surface approaches. Though, both factors do have clearly identified motive and strategy sub-components which may be of interest as well [1].

The R-SPQ-2F was criticized by Justicia, Pichardo, Cano, Berbén and De la Fuente however, not only with respect to the way in which the questionnaire was developed, but also with regard to the dimensionality posited [5]. In an empirical study, they collected data for the R-SPQ-2F from two independent samples of Spanish students (n = 314 first year students of education with a mean age of 20 and n = 522 final year students of education and psychology with a mean age of 23, respectively). The R-SPQ-2F was translated into Spanish and modified where necessary in order to take cultural differences into account. Factor analysis did provide empirical support for the two-factor structure; however it did not provide empirical support for the four-factor structure. No evidence was found to support the differentiation between motive and strategy sub-components. Justicia et al. concluded that this brings a new perspective on the underlying structure of the R-SPQ-2F, i.e. that only computing main scale scores makes sense [5].

Fryer, Ginns, Walker, and Nakao tested whether a translation of the R-SPQ-2F into Japanese is a valid measure of students’ approaches to learning within the Japanese tertiary context [6]. Discussions with two focus groups of students (n = 10 and n = 12, respectively) of mixed major (Management and Commerce) second-year students at a Japanese four-year tertiary institution suggested that the wording of all items was clearly. However, some of the items originally meant to describe a surface approach, were not clearly representing a surface approach according to the students. Following this qualitative pilot study, data for the R-SPQ-2F were collected from 273 mixed major (Management and Commerce) second-year students at the same Japanese institution. Factor analysis did provide empirical support for the four-factor structure. However, the reliabilities of the four sub-scales were unsatisfactory. While the reliability of the Deep Approach main scale was good (Cronbach’s alpha = 0.76), the reliability of the Surface Approach main scale was marginal (Cronbach’s alpha = 0.60) but equivalent to the reliability reported by Biggs, Kember, and Leung. Remarkably, a positive correlation (r = 0.30) was found between the Deep and Surface main scales. Fryer et al. concluded that their results did not indicate that Deep and Surface approaches were composed by Japanese students in the same manner as students in other countries [6]. Especially, the Surface Approach scale appeared to be less valid [6].

Immekus and Imbrie tested the factor structure of the R-SPQ-2F among Western incoming freshmen students attending a university in the United States (cohort 1: n = 1490, 289 females and 1201 males; cohort 2: n = 1533, 297 females and 1236 males) [7]. Factor analysis on the basis of the data of cohort 1 did not provide empirical support for the original two-factor structure neither for the four-factor structure. Instead, an alternative four-factor model was supported and some items were deleted. Remarkably, the alternative four factors resembled those reported by Biggs, Kember, and Leung. Factor analysis on the basis of the data of cohort 2 cross-validated the results of the first cohort study. Immekus and Imbrie concluded that their findings suggested the R-SPQ-2F-structure being cross-culturally sensitive, although the emergence of Deep and Surface factors across culture groups seems to be supported [7]. Continued research is needed to determine the extent to which scores on the R-SPQ-2F represent the underlying constructs when obtained from students with different cultural backgrounds [7].

Leung, Ginns, and Kember tested whether approaches to learning as measured by the R-SPQ-2F were consistent in Western and Eastern university contexts [8]. Participants in the study were undergraduate and postgraduate students from one university in Sydney and two universities in Hong Kong. In the Hong Kong sample, about half of the undergraduates were Year 1 students and the other half of the sample consisted of 24% Year 2 students and 26% Year 3 students. In the Australian sample, the undergraduate students were enrolled in Year 1 (36%), Year 2 (25%), Year 3 (23%), Year 4 (13%) and Year 5 (3%). In both samples, students were enrolled in a wide range of disciplines [8]. The research results suggested that the same characterization of approaches to learning can be used in both contexts. The continuum from Surface to Deep Approaches is applicable in both contexts. University students from Sydney and from Hong Kong both use the same conceptual frame of reference when making responses to the R-SPQ-2F. However, Hong Kong students score higher on both Deep and Surface approaches than the Australian students; they use intermediate approaches to a greater extent. Leung, Ginns, and Kember conclude further research into the cross-cultural specificity of approaches to studying is needed [8].

The present study investigated whether the structure of the R-SPQ-2F as posited by its original authors is found when a translated version is used in higher education in a Dutch context. By comparing our results with the results of earlier studies into the validity and reliability of the R-SPQ-2F in different cultures, the cross-cultural sensitivity of the questionnaire is discussed.

### Research Questions and Methods

#### Research Questions

In this study, we investigated whether the positive results yielded by analyses into the validity and reliability of the original English version of the R-SPQ-2F [1] would also apply to the Dutch version which we developed. Development and evaluation of psychometric properties of the R-SPQ-2F were based on a sample of Hong Kong university students [1]. Recent studies by Fryer et al. [6], Immekus and Imbrie [7], and Justicia et al. [5] after all, revealed the cross-cultural sensitivity of the R-SPQ-2F and questioned the cross-cultural validity of the R-SPQ-2F as a means of obtaining a picture of a student’s approach to studying. Leung, Ginns, and Kember suggested further research into the cross-cultural specificity of approaches to studying is needed [8]. Our first research question was thus: “Is the R-SPQ-2F a cross-culturally sensitive instrument?” Our second research question was: “Is the Dutch version of the R-SPQ-2F sufficiently valid and reliable for measuring the approach to studying of a student in higher education in a Dutch context?”.

### Instrument

The R-SPQ-2F is a brief questionnaire which is simple to use. The current version consists of 20 items. The items are scored on a five-point Likert scale ranging from Seldom/Never True to Always/Almost Always True. The questionnaire gives an indication of how a student believes that he or she approaches studying. A distinction is made between a surface approach to studying, intended to meet the requirements of courses with a minimum effort, and a deep approach to studying whereby the student has an intrinsic interest in studying and enjoys studying.

We developed a Dutch version of the R-SPQ-2F, which was a translation of the 20 original items with minor wording adjustments to fit the context of higher education in Flanders (Belgium). To this end, we began by following the methodology described by Lindblom-Ylänne, Trigwell, Nevgi, and Ashwin [9]: two researchers, who were not involved either in the creation of the Dutch version of the R-SPQ-2F or in this validation study, translated the items from the Dutch version back into the English. We were then able to use this back translation to detect slight differences in formulation between the translated and the original versions. The items concerned underwent slight wording modifications in our final Dutch version. Care was taken to ensure equivalence rather than literacy in translation. It should be noted that Biggs, Kember, and Leung themselves recommend that the formulation of the items in the questionnaire should be tailored as closely as possible to the particular course in question [1]. So we formulated all items course-specific and in the past tense. For example an original item as ‘I find that at times studying gives me a feeling of deep personal satisfaction’ became ‘Studying for this course gave me at times a feeling of deep personal satisfaction’.

### Respondents

Within the framework of a larger study into the impact of teacher training on students’ learning, the Dutch version of the R-SPQ-2F was offered to 2023 students at the University of Antwerp (Belgium). In all, 1974 students returned a fully completed questionnaire (response rate of 98%). The students were enrolled in a wide range ofacademic disciplines. 28% of our sample were belonging to a ‘pure hard’ discipline [10] such as chemistry; 30% were belonging to an ‘applied hard’ discipline [10] such as medicine; 11% were belonging to a ‘pure soft’ discipline [10] such as history, and 29% to an ‘applied soft’ discipline [10] such as education. For 2% of the respondents the discipline was unknown.40% of our sample were males. 59% were first-year students; the other 41% were students across all other years of study. Mean age was 21 (minimum 18, maximum 47).

Taking into account these characteristics of our respondents, we conclude our sample is comparable regarding discipline to the sample used in the study by Biggs, Kember, and Leung [1] as well as to the sample used by Leung, Ginns, and Kember [8]. The samples in the studies by Justicia et al. [5] and Fryer et al. [6] were more defined in terms of discipline. Only students of education and psychology, and of management and commerce, respectively, were involved in their studies. In the article about the study of Immekus and Imbrie [7] no information was found about the discipline(s) of the respondents. Regarding gender our sample differs from the sample used by Immekus and Imbrie [7], as in their study 81% males were involved (in cohort 1 as well as in cohort 2). In the articles about the other reference studies (Biggs, Kember, and Leung [1]; Leung, Ginns, and Kember [8]; Justicia et al. [5]; Fryer et al. [6]) no information was found about the gender of the respondents. Regarding level of study our sample is comparable to the sample used in the study by Biggs, Kember, and Leung [1]. It is comparable regarding level of study to the sample used by Leung, Ginns, and Kember as well [8], besides that in their study postgraduate students were involved too. Justicia et al. [5], Fryer et al. [6], and Immekus and Imbrie [7] used a more defined sample in terms of educational level: they only involved first year and final year students, second-year students, and first-year students, respectively. Regarding age our sample differs a bit from the samples used by Justicia et al. [5], as in their two samples mean ages were 20 and 23, respectively. In the articles about the other reference studies (Biggs, Kember, and Leung [1]; Leung, Ginns, and Kember [8]; Immekus and Imbrie [7]; Fryer et al. [6]) no information was found about the age of the respondents.

### Analysis of the Data

We subjected the data to a confirmatory factor analysis, in which we looked at whether the two-factor structure for which Biggs, Kember, and Leung found evidence [1] also fitted our data. As this did not appear to be the case, we then carried out a maximum likelihood factor analysis with oblique rotation on a (randomly selected) half of the data. The model that we posited on the basis of the results of this maximum likelihood factor analysis was then subjected to a confirmatory factor analysis on the other half of the data.

In order to check the fit of the confirmatory factor structures we used a variety of different indices: the goodness-of-fit index (*GFI*); the adjusted goodness-of-fit index (*AGFI*); the comparative fit index (*CFI*), the Root Mean Square Error of Approximation (*RMSEA*), the parsimony goodness-of-fit index (*PGFI*), the parsimony comparative fit index (*PCFI*), the Akaike’s Information Criteria (*AIC*), the Bayesian Information Criteria (*BIC*) and the number of standardized residuals labelled as problematic, that is >2.58 [12]. *GFI*, *AGFI* and *CFI* values equal to or greater than 0.90 and an *RMSEA* value equal to or smaller than 0.05 were taken as an indication that the data showed a relatively good fit with the model [11]. Regarding *PGFI*, *PCFI*, *AIC*, *BIC* and the number of standardized residuals labelled as problematic there are no cut-off criteria as they are sensitive to model size; their values were used to compare models [11]–[13]. As a reference, it is often said that the sample has to be of a size between 200 and 500 observations in order that the chi-squared test can be used to check the goodness-of-fit [11], [14]. This test was therefore not suitable for the number of respondents that we had.

In our exploratory maximum likelihood factor analysis the number of factors was determined on the basis of interpretability and Horn’s method [15]. We also opted for obliquely rotated solutions because this not only made the interpretation of the factors easier, but also starts from the principle that factors might correlate. In interpreting the maximum likelihood factor analysis we did not take into account loadings of items between −.40 and.40.

## Results

As a first step we used a confirmatory factor analysis (CFA) to examine the dimensionality of the R-SPQ-2F in more detail. The confirmatory factor analysis explored the two-factor structure (Deep Approach-Surface Approach) for which Biggs, Kember, and Leung [1] found evidence. The fit indices for this two-factor model are given in Table 1, from which it can be seen that this model does not fit the data well (GFI = 0.86, AGFI = 0.82, CFI = 0.80, RMSEA = 0.09, PGFI = 0.69, PCFI = 0.71, AIC = 2845.39, BIC = 3074.49, number of residuals >2.58∶71 out of 210).

**Table 1:** Goodness of Fit Indices for CFA for the Two-Factor Model According to Biggs, Kember, and Leung (2001) and for Our Measurement Models.

Index	2-Factormodel	Measurementmodel accordingto results FA	Measurement model according to results FA leaving out the sub-scale Self-regulated Learning	Measurement model according to results FA leaving out the sub-scale Self-regulated Learning and with item 7 related to 2 factors	Measurement model according to results FA leaving out the sub-scale Self-regulated Learningand item 7	Final measurement model as shown in Figure 1
GFI	0.86	0.89	0.93	0.94	0.95	0.95
AGFI	0.82	0.86	0.90	0.92	0.93	0.93
CFI	0.80	0.86	0.90	0.93	0.94	0.94
RMSEA	0.09	0.08	0.07	0.06	0.06	0.06
PGFI	0.69	0.69	0.67	0.68	0.67	0.66
PCFI	0.71	0.74	0.75	0.76	0.76	0.75
AIC	2845.39	1124.47	724.23	554.62	485.74	470.33
BIC	3074.49	1340.89	906.22	741.54	657.90	647.40
Number of residuals>2.58	71 out of 210	53 out of 190	19 out of 136	8 out of 136	7 out of 120	4 out of 120

Given that our data did not show a good fit with the two-factor model, we carried out a maximum likelihood factor analysis (FA) with oblique rotation. Because we wanted to subject the model that we posited on the basis of the results of this maximum likelihood factor analysis to a confirmatory factor analysis, only the data of (a randomly selected) half of the students who filled in all questionnaire items (n = 963) was used. Five underlying factors could be identified (Table 2). A total of 59.87% of the item variance can be explained by these five underlying factors. The first factor, Studying Is Interesting, measures the extent to which students state that virtually any topic is interesting and that they work hard because they find the material interesting. The second factor can be called Learning By Heart, and measures the extent to which students regard trying to remember answers to likely questions as the best way to pass the assessment and learn some things by rote even without understanding them. The third factor, Spending Extra Time On Studying, measures the extent to which students state that they spend a lot of free time on finding out more on topics discussed in class and on looking at the suggested readings for the course. We can interpret the fourth factor as Studying With As Less Effort As Possible. This factor measures the extent to which students only study the material given in class or in the outline and see no point in learning material which is not likely to be assessed. The final factor is what we have called Self-regulated Learning and measures the extent to which students test their selves and state they want to do much work in order to be able to form their own conclusions concerning the topics of the course.

**Table 2:** Loadings of Items, Eigenvalue and Percentage of Explained Variance for the Dimensions of the Dutch Version of the R-SPQ-2F for Maximum Likelihood Factor Analysis with Oblique Rotation (Loadings between −0.40 and 0.40 omitted).

Item	Loading				
	M1	M2	M3	M4	M5
i5	0.84				
i13	0.55				
i7	−0.45				
i1	0.44				
i11		0.69			
i20		0.66			
i8		0.64			
i14			−0.77		
i6			−0.66		
i18			−0.60		
i9			−0.40		
i17					
i19				0.75	
i16				0.73	
i12				0.50	
i15				0.48	
i4				0.47	
i3					−0.46
i10					0.40
i2					0.39
Eigenvalue	5.83	2.72	1.31	1.09	1.03
Percentage explainedvariance	29.16	13.58	6.57	5.43	5.13
Cumulative percentageexplained variance	29.16	42.74	49.31	54.74	59.87

All items loaded on one factor, except item 17 (“I came to most classes with questions in mind that I wanted to be answered”) loading sufficiently on none of the factors. Item 2 (“I found that I had to do enough work for this course so that I could form my own conclusions”) loaded nearly.40 on the factor Self-regulated Learning. As this loading was explicable in relation to content we decided to retain item 2 in our further analyses.

We note that the factors Studying With As Less Effort As Possible and Learning By Heart on the one hand relate to the Surface Approach factor in the two-factor model as identified by Biggs, Kember and Leung [1]. The other three factors are related to the Deep Approach factor. For this reason, in a subsequent step, we carried out a confirmatory factor analysis on a model with two main factors (Surface Approach and Deep Approach) and five sub-factors. The sub-factors Minimal Effort and Learning By Heart are related to the main factor Surface Approach; the sub-factors Studying is Interesting, Spending Extra Time, and Self-regulated Learning are related to the main factor Deep Approach.

Table 3 shows the Cronbach alpha values for both the main factors and sub-factors. The internal consistency of the main factors, calculated on the basis of the underlying items, is good: Deep Approach (α = 0.84), and Surface Approach (α = 0.81). Four sub-scales appear to be sufficiently internally consistent: Studying Is Interesting (α = 0.80), Spending Extra Time (α = 0.79), Minimal Effort (α = 0.78), and Learning By Heart (α = 0.73). The sub-scale Self-regulated Learning (α = 0.47) appears to be insufficiently reliable and cannot be used in further analyses. Table 3 also shows the mean scale scores and corresponding standard deviations. The standard deviations indicate that inter-individual differences exist with regard to scores on the different scales and sub-scales. The questionnaire thus has a certain discriminatory capacity with regard to ascertaining the approach to studying of individual students.

**Table 3:** Cronbach’s α-values, Means and Standard Deviations for the Scales and Sub-Scales in the Measurement Model according to results FA.

(Sub)scale	Cronbach’s α	Mean	Standard deviation
Deep approach	0.84°	2.68	0.68
Surface approach	0.81°	2.16	0.76
Studying is interesting	0.80	3.04	0.90
Spending extra time	0.79	1.83	0.80
Self-regulated learning	0.47	3.33	0.79
Minimal effort	0.78	2.40	0.88
Learning by heart	0.73	1.78	0.87

°Calculated on the basis of the underlying items.

We subjected the model as posited to a confirmatory factor analysis. The goodness-of-fit-indices are shown in Table 1 (GFI = 0.89, AGFI = 0.86, CFI = 0.86, RMSEA = 0.08, PGFI = 0.69, PCFI = 0.74, AIC = 1124.47, BIC = 1340.89, number of residuals >2.58∶53 out of 190). Based on these indices, we concluded that our data show an insufficient fit with the model.

In a next step, we repeated the confirmatory factor analysis leaving out the sub-scale Self-regulated Learning as this sub-scale revealed to be unreliable. The goodness-of-fit-indices are shown in Table 1 (GFI = 0.93, AGFI = 0.90, CFI = 0.90, RMSEA = 0.07, PGFI = 0.67, PCFI = 0.75, AIC = 724.23, BIC = 906.22, number of residuals >2.58∶19 out of 136). Based on these indices, we concluded that our data show a better fit with this model. However, we did remark that most of the problematic residuals concerned item 7 (“I didn’t find this course very interesting so I kept my work to the minimum”). We tried two solutions. The modification indices indicated that relating item 7 not only on the factor Studying Is Interesting but on the factor Studying With As Less Effort as well, would improve the model. As this was explicable in relation to content (the first half of the item refers to experiencing studying as something interesting; the second half centres on doing as little study work as possible), we decided to relate item 7 on both factors in order to see whether this would be a solution. As a second solution we removed item 7.

We first subjected the model with item 7 loading on two factors to a confirmatory factor analysis. The goodness-of-fit-indices are shown in Table 1 (GFI = 0.94, AGFI = 0.92, CFI = 0.93, RMSEA = 0.06, PGFI = 0.68, PCFI = 0.76, AIC = 554.62, BIC = 741.54, number of residuals >2.58∶8 out of 136). Based on these indices, we concluded that our data show a good fit with the model. However the model without item 7 showed a still better fit (GFI = 0.95, AGFI = 0.93, CFI = 0.94, RMSEA = 0.06, PGFI = 0.67, PCFI = 0.76, AIC = 485.74, BIC = 657.90, number of residuals >2.58∶7 out of 120).

When considering the standardized weight factors for this latest model we remarked the weight of the sub-factor Studying Is Interesting on the factor Deep Approach as well as the weight of the sub-factor Minimum Effort on the factor Surface Approach being larger than 1 (1.05 and 1.12 respectively). This indicates a Heywood case [16] and therefore the model being not suitable.

**Figure 1:**
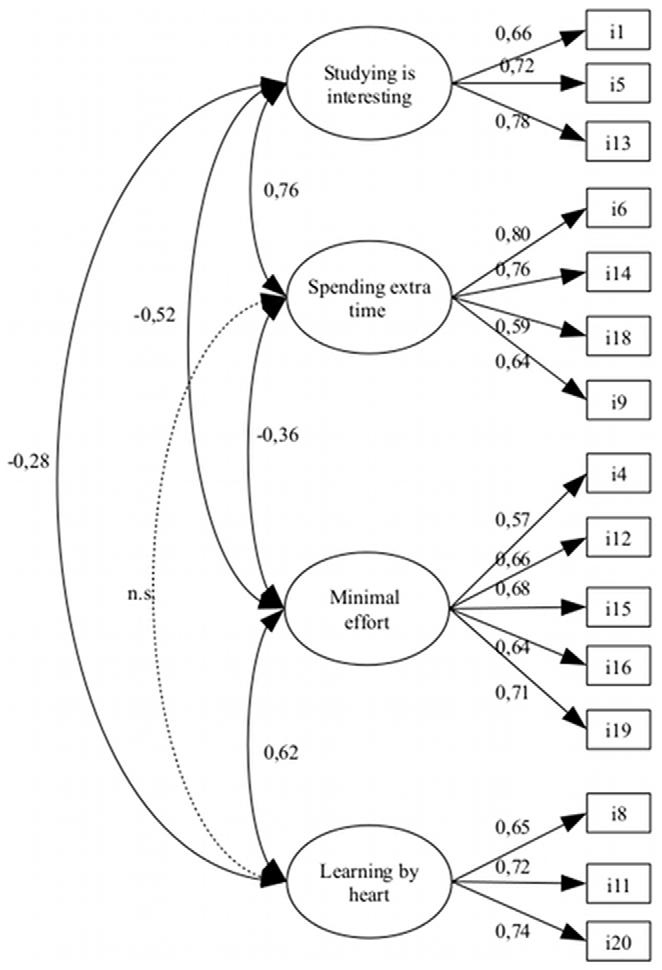
Factor Structure based on the best fitting CFA model.

Given the indication of a Heywood case, we checked whether a new confirmatory model with 4 first-order factors presented proper fit. The goodness-of-fit-indices are shown in Table 1 (GFI = 0.95, AGFI = 0.93, CFI = 0.94, RMSEA = 0.06, PGFI = 0.66, PCFI = 0.75, AIC = 470.33, BIC = 647.40, number of residuals >2.58∶4 out of 120). Based on these indices, we concluded that our data show a good fit with the model. Figure 1 shows the model that we posit.

Finally, we used the correlation coefficients based on the confirmatory factor analysis to investigate the underlying correlations between the scales in the model (Table 4).

**Table 4:** Correlation Coefficients based on the CFA for the Scales in the Final Measurement Model.

	Studying is interesting	Spending extra time	Minimal effort	Learning by heart
Studying is interesting	1.00	0.76***	−0.52***	−0.28***
Spending extra time		1.00	−0.36***	−0.07
Minimal effort			1.00	0.62***
Learning by heart				1.00

*** *p*<.001.

## Discussion

In the present study, a confirmatory factor analysis for the Dutch version of the R-SPQ-2F did not show a good fit of our data, collected from 1974 students in university education in Flanders (Belgium), with the two-factor model for which Biggs, Kember, and Leung [1] found evidence. In answer to our first research question, the results of our study suggest that the R-SPQ-2F is a cross-culturally sensitive instrument. It appears that the posited structure of the questionnaire is not automatically found when a translated version is used in the Dutch context. Likewise Fryer et al. [6], Immekus and Imbrie [7], and Justicia et al. [5], in their analysis of data collected in Japan, the United States, and Spain respectively, did not find evidence for the dimensionality as posited by the authors of the R-SPQ-2F. Cross-cultural sensitivity is therefore an important point to be borne in mind when using the questionnaire. The cross-cultural sensitivity of the questionnaire might result from different meanings attached to a single word or concept, e.g. because students are operating in different higher education systems. Higher education in Flanders (Belgium), for example, is characterised by no pre-selection of students; the entrance into higher education is very open. The selection of students capable for study takes place during higher education and so the assessment of students during courses is (at least partly) selection-oriented. This might influence the way in which students interpret questionnaire items concerning assessment.

As in the studies by Fryer et al. [6], Immekus and Imbrie [7], and Justicia et al. [5], our study revealed that it is important to avoid automatically fitting the results obtained with the R-SPQ-2F questionnaire into the factor structure postulated by its authors. One of the possibilities for future research might be to repeat Biggs’ own development study in culturally different contexts, thereby taking into account the concerns indicated by Justicia et al. regarding rigour and methodology [5]. This could be combined –as in the recent study by Fryer et al. [6]- with qualitative research into the possible different meanings which can be attached to a single concept.

In the present study, in the context of higher education in Flanders (Belgium), maximum likelihood factor analysis with oblique rotation and confirmatory factor analyses indicate that our data show best fit with the model shown in Figure 1. When we compare the factors from our model with the factors from Biggs, Kember and Leung’s four-factor model, we observe that all items of the factor Spending Extra Time belong to the factor Deep Strategy in the four-factor model, with the exception of item 9 belonging to the factor Deep Motive. The items of the factor Studying Is Interesting all belong to the factor Deep Motive in the four-factor model. The factor Minimal Effort contains both items which, in the four-factor model, are subsumed under the factor Surface Strategy, as well as items which, in that model, belong to the factor Surface Motive. The same applies to the factor Learning By Heart from our model.

Just like the findings of Immekus and Imbrie [7], our findings too, support an alternative model resembling though the factors of the four-factor model reported by Biggs, Kember, and Leung [1]. Like Immekus and Imbrie [7] we conclude the R-SPQ-2F-structure being cross-culturally sensitive, although the emergence of Deep Motive, Deep Strategy and Surface factors across culture groups seems to be supported. No clearly identified Surface Motive and Surface Strategy factors were found. This is in line with the findings of Fryer et al. revealing the Surface Approach Scale being less valid in the Japanese tertiary context [6]. It is consistent with the suggestion of both Kember and Gow [17] and Richardson [18] that surface study approaches are less culturally portable than deep study approaches which are broadly intercultural agreed as suiting the purpose of higher education. Our data don’t give evidence for the criticism of Justicia et al. that only computing main scale scores makes sense [5].

The sub-scale Self-regulated Learning revealed to be insufficiently reliable (α = 0.47) and was not retained in our final model. Here too, the cross-cultural sensitivity of the questionnaire might have played a role and the explanation could lie in the culture of higher education in the Dutch context, where ‘forming one’s own conclusions concerning the course topics’ (Item 2), ‘testing oneself’ (Item 10), and ‘aiming to pass the course while doing as little work as possible’ (Item 3) are perhaps not seen as aspects of the same construct. Wider use of the Dutch version of the R-SPQ-2F is desirable in order to investigate this interpretation further.

On the basis of our validation study our second research question can be answered affirmatively. It appears that the Dutch version of the R-SPQ-2F is sufficiently valid and reliable to use when the objective, as part of educational research or education practice, is to ascertain the approach to studying of students in higher education in the Dutch context. It can be used to analyse relationships between the approach to studying and other aspects in the teaching and learning environment (the teaching approach of the teacher or the way the assessment is organized) [1]. It is also useful in establishing the effect of educational innovations in teaching environments on students’ approach to studying [1], [19]. In this connection, a limitation of the questionnaire relates to the fact that it basically provides quantitative data. The additional use of qualitative data, perhaps obtained via interviews with the students concerned or via documentary analysis, also might offer the possibility of triangulation and greater depth and, in so doing, make a significant contribution.

Follow-up research might help to further confirm the validity and reliability of the Dutch version of the R-SPQ-2F or to optimise the questionnaire with a view to its more widespread use. In our present study, we did not set out to give a representative picture of the approach to studying of the average student in higher education in Flanders. We remark that representing such a picture would have been even impossible because the R-SPQ-2F is a relational inventory: it does not assess general orientations but specific responses to particular situations [1]. The data which the questionnaire generates are very context-bound: if a student were to complete the questionnaire for a different course or another study context, his or her score might well be very different. When interpreting the data, it is therefore important that elements relating to this context (mandatory course or not, number of students following the course, basic course or follow-up course) are also taken into account. Context data of this kind can often be obtained from the teaching administration department of the institution in which the questionnaire is administered. If not, it is important to secure this information from the respondents themselves when conducting the R-SPQ-2F.
